# A biomechanical study of load carriage by two paired subjects in response to increased load mass

**DOI:** 10.1038/s41598-021-83760-6

**Published:** 2021-02-23

**Authors:** Guillaume Fumery, Nicolas A. Turpin, Laetitia Claverie, Vincent Fourcassié, Pierre Moretto

**Affiliations:** 1 Centre de Recherches Sur La Cognition Animale, Centre de Biologie Intégrative, Université de Toulouse, CRCA, UMR CNRS-UPS 5169, 118 Route de Narbonne, 31062 Toulouse, France; 2 Physical Medicine and Rehabilitation Center, MAS Marquiol, Toulouse, France; 3 IRISSE Lab (EA 4075), UFR SHE, Sport Sciences Department (STAPS), Université de La Réunion, 117, rue du général Ailleret, 97430 le Tampon, France

**Keywords:** Mechanical engineering, Bioenergetics, Behavioural ecology, Patient education

## Abstract

The biomechanics of load carriage has been studied extensively with regards to single individuals, yet not so much with regards to collective transport. We investigated the biomechanics of walking in 10 paired individuals carrying a load that represented 20%, 30%, or 40% of the aggregated body-masses. We computed the energy recovery rate at the center of mass of the system consisting of the two individuals plus the carried load in order to test to what extent the pendulum-like behavior and the economy of the gait were affected. Joint torque was also computed to investigate the intra- and inter-subject strategies occurring in response to this. The ability of the subjects to move the whole system like a pendulum appeared rendered obvious through shortened step length and lowered vertical displacements at the center of mass of the system, while energy recovery rate and total mechanical energy remained constant. In parallel, an asymmetry of joint moment vertical amplitude and coupling among individuals in all pairs suggested the emergence of a leader/follower schema. Beyond the 30% threshold of increased load mass, the constraints at the joint level were balanced among individuals leading to a degraded pendulum-like behavior.

## Introduction

Load carriage is a common yet complex task for human beings. It has been studied in various domains, including anthropology (e.g. African porters^1^, Himalayan porters^2^), ergonomics (e.g. nursing^3^, transporting military equipment^4,5^ or carrying school bags^6^), sports (e.g. weightlifting^7^), and rehabilitation (e.g. pregnancy^8^).

These studies have substantially improved our understanding of the biomechanical consequences of carrying a load in terms of locomotor pattern or energetic costs. For example, they have shown that the recovery rate -quantifying the transfer of energy between kinetic and potential energy at the center of mass (CoM) of the body, due to the pendulum-like behavior of the CoM during walking^9^-decreases when walking with a load, but can increase again with experience or training ^4,5^. Moreover, it has been reported that increasing the load mass in single carriers reduces the vertical amplitude of the CoM trajectory and the duration of strides, while increasing step length^1,2^.

An equally common, but less studied load carriage task is the collective transport of loads by two or more individuals. The contexts in which this task is performed are numerous. Collective transportation arises generally every time a load is too heavy, or an object too large, to be handled by a single subject. In addition to coping with the constraints associated with the load itself, it requires coordination among individuals while walking. Only a few studies have focused on this, and it is not clear yet whether the results obtained concerning single carriers can be extrapolated to cooperative transportation^10,11^. For example, one does not know whether the strategies used to optimize the energetic cost or to limit musculoskeletal constraints differ between individual and collective load carrying.

We have shown in two previous studies that the gait pattern of the system formed by a pair of individuals carrying a load together is not affected by light loads amounting to ~ 10% of the sum of their body masses^10,11^. These studies thus demonstrated that the gait pattern of this system is as economical in terms of external energy, as the gait pattern of individuals walking alone with the same load. This would suggest that when two individuals are carrying a load together they are not mutually influenced, and therefore that the results obtained from single carriers could be extrapolated to individuals participating in a collective transport task. Nevertheless, it is also possible that the load used in these experiments was not heavy enough to induce significant changes in the locomotor patterns of each individual. The main objective of the present study was therefore to investigate to what extent the mass of a load carried by two individuals affects their locomotor pattern and the economy of walking. Based on previous studies on single individuals^1,4,12^, we hypothesized that increasing loads should lead to a decrease in the pendulum-like behavior of the CoM and the recovery rate, to a decrease in the velocity and vertical amplitude of the center of mass of the system formed by the two individuals and the load they carry, as well as to a decrease in the step length of individuals. Moreover, based on our previous study on collective load transport, we also hypothesized that increasing loads above 10% of the total load mass should induce an increase in the total mechanical energy of the system. We consequently computed all these parameters in our study.

In biomechanics literature, the computation of the external and total energy of the body is often restricted to the center of gravity of the subjects. This is a problem, however, because in so doing the energy due to the movements of body segments is ignored—i.e. the internal mechanical energy linked to the work of the muscles—and this can alter results considerably. When two subjects collaborate in carrying a load, the internal mechanical energy and power can differ from one subject to the other depending on the motor strategies each of them develops, and how each of them contributes to the task. To address these differences across the paired subjects, we performed a detailed biomechanical analysis in which we considered the two subjects and the load they carry as a poly-articulated system, i.e. a system in which all the segments are linked by frictionless joints. We estimated the internal energy and power of this system, as well as the forces and joint moments developed at the upper limbs, shoulders, neck and back levels of the subjects. To our knowledge, this is the first time this method is used to estimate the mechanical cost of a collaborative task exhaustively by taking into account all the segments of a system. This biomechanical analysis also allowed us to investigate the strategies used by individuals to perform the load carriage task together. With this aim in view, we identified co-variation patterns across the different joint moments of each individual in the same pair considered separately, and across the joint moments of the two individuals in the same pair considered as a whole. This analysis enabled us to group the joint moments into functional units, called dynamic synergies^13^, and to reveal the coordination structure within each individual, as well as across individuals. The analysis of these synergies led to identify regularities and variabilities reciprocally in the spatial or temporal patterns of joint torque, joint kinematic or muscle activation, within subjects or across several subjects^14–18^. The analysis is particularly useful to gather insights about the motor strategies put in place by the central nervous system to cope with the different task constraints^17,19,20^. Moreover, no previous studies analyzed arm synergies during a collective task.

Overall, we conducted this study with the aim of testing whether the motor behavior of two subjects carrying a load together obeys the same optimization principle as that of single carriers. In this respect, we attempted to answer the three following questions (i) does the pendulum-like behavior of the CoM typically observed in single-subject locomotion persist at the level of the system formed by two individuals and the load they carry? (ii) do the mechanical cost and power stay stable as long as the mass of the load is below a certain threshold?, (iii) can the intra and inter-subject synergies reveal a strategy adopted by the subjects to collaborate in the carrying task?

In view of the results obtained regarding the energy invested in transport, the way the subjects organized or not their collaboration, the motor strategies they developed**,** and the resulting constraints at the joint, our study could prove very useful in the fields of robot-human interactions and cobotics, as well as in ergonomics and physical education training aimed at preventing pain and injuries.

## Results

**Figure 1:**
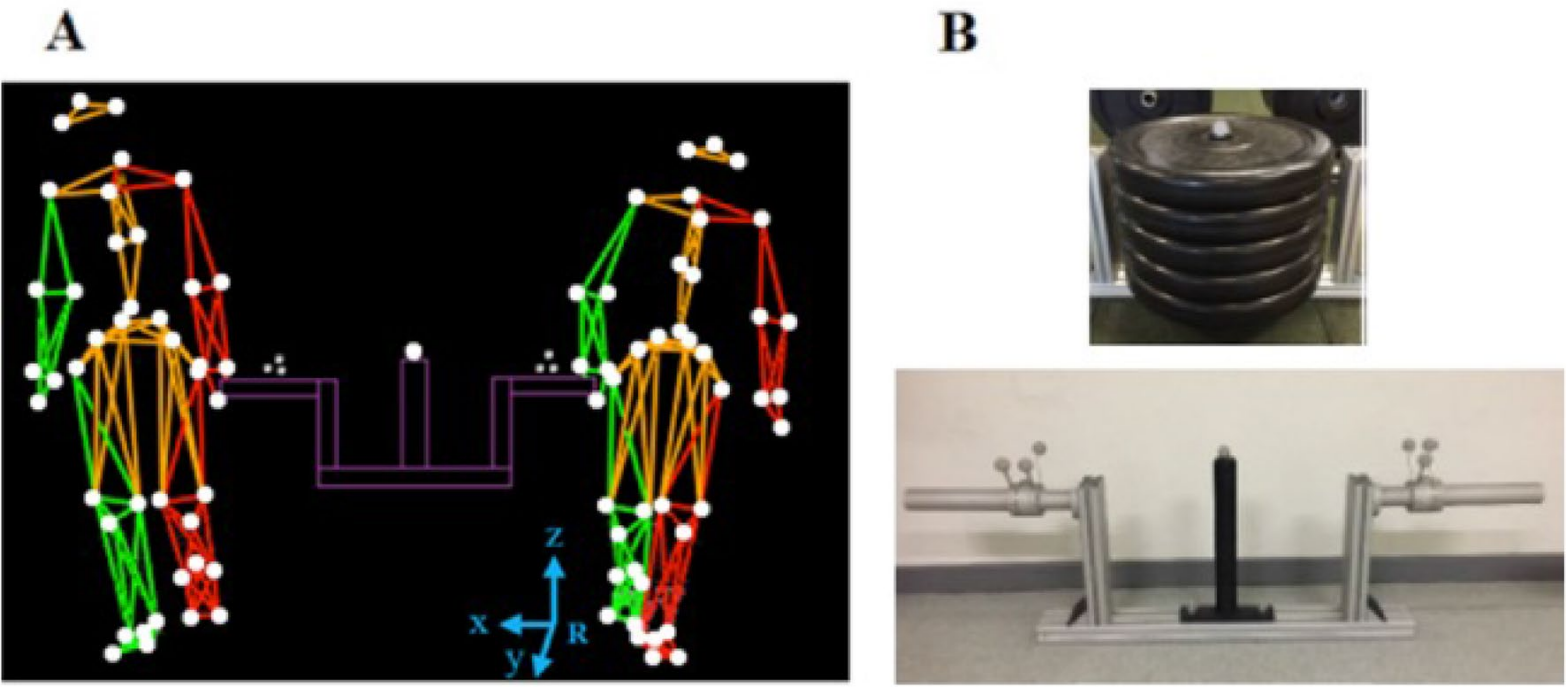
Collective load transport task and carried object. The pairs of volunteers (**A**) had to walk a distance of 20 m along a straight line while collectively carrying an object. Depending on experimental condition, the mass of the object they carried represented 20%, 30% or 40% of the sum of the body mass of the two volunteers. The white dots on the image correspond to kinematic markers. The kinematic model as reconstructed by the Vicon Nexus™ software is represented in red for the left side, in green for the right side, in orange for the head, trunk and pelvis, and in purple for the load. The global coordinate system (R) is indicated in the lower part of the figure, with *x* as medio-lateral axis, *y* as antero-posterior axis, and *z* as vertical axis. The carried object (**B**) was of the following dimensions: 1.16 × 0.45 × 0.37 m, and was equipped with force sensors at the handles. It comprised a rod at its center around which standard cast iron discs could be slid to increase its weight.

A total of twenty individuals divided into ten pairs participated in the study. Nevertheless, because of the loss of one reflective marker in one pair, only nine pairs were eventually analyzed. The two individuals composing each pair walked side by side while carrying the load with one hand. The load was held with a pronation grasp using a handle fixed on each side (Fig. 1). Its weight represented 20%, 30% or 40% of the sum of the body masses of the two individuals and it thereafter referred to three collectives transport (CT) conditions as CT20, CT30, and CT40 respectively. In our analysis we considered the two individuals and the load they carry as a whole, i.e., as a Poly-Articulated Collective System (PACS)^21^. The individuals were instructed to carry the load from one side of the experimental room to the other. In order for behaviors to be as spontaneous as possible, no other instructions were provided.

**Figure 2:**
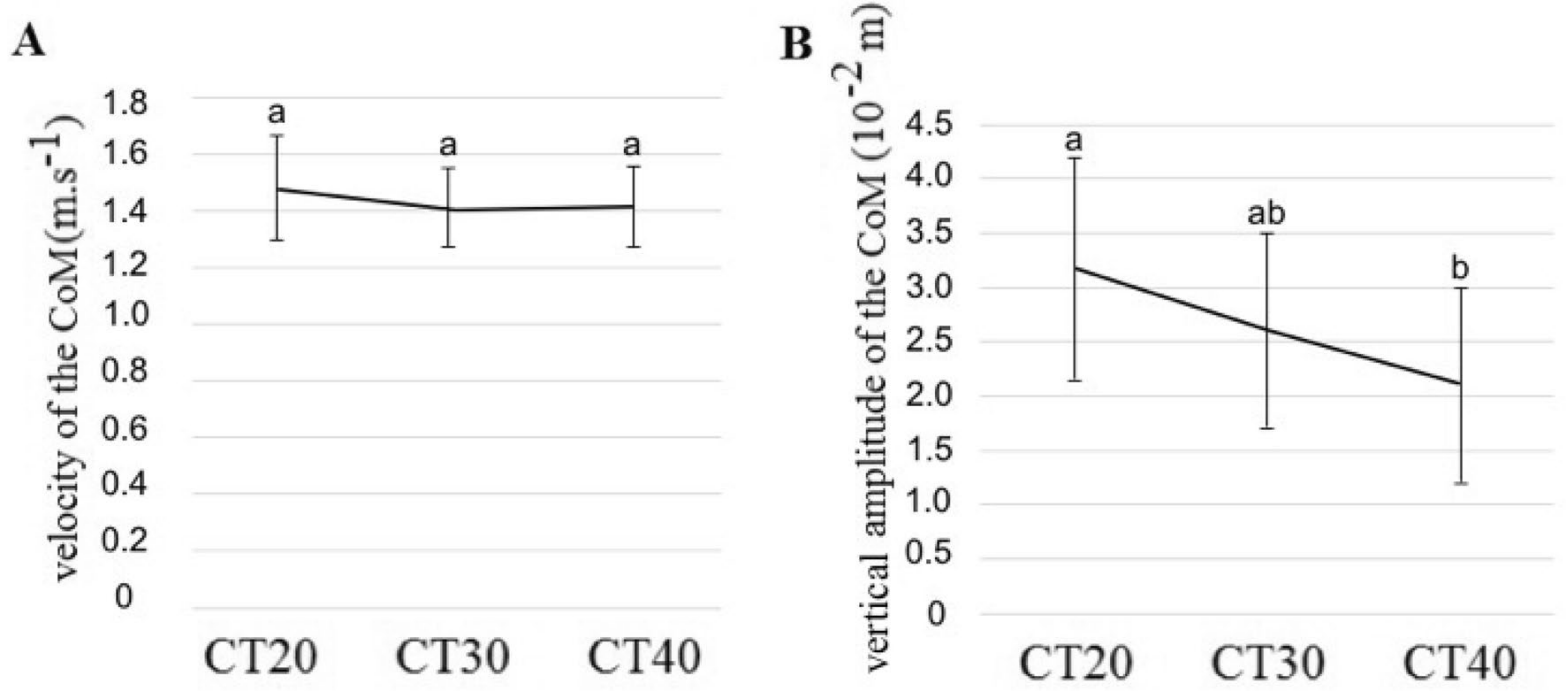
Velocity and vertical amplitude of the displacement of the center of mass (CoM) of the system formed by the two individuals and the load they carry. Mean (± SD) of the velocity (**A**) and vertical amplitude (**B**) of the vertical displacement of the system formed by the two individuals plus the box they carry (*N* = 9 pairs). The three conditions CT20, CT30, and CT40 differed in terms of the transported mass, corresponding to 20%, 30%, and 40% of the sum of the body masses of the paired participants, respectively. For the same variable, the bars bearing different letters represent significantly different values (GLMM followed by a *post-hoc* test for multiple comparisons across conditions).

The velocity of the PACS center of mass (CoM_PACS_) was not affected by the load (Fig. 2A, χ^2^ = 4.18, *P* = 0.12). The duration of the PACS’ gait cycle was not mass sensitive (χ^2^ = 4.55, *P* = 0.17; 1.09 ± 0.11, 1.12 ± 0.07, and 1.08 ± 0.16 s in the CT20, CT30, and CT40 conditions respectively). However, the vertical amplitude of the CoM_PACS_ trajectory decreased by 32.85% between CT20, and CT40 (Fig. 2B, z = − 2.65, *P* = 0.02). The length of the subject’s gait cycles, corresponding to two steps, decreased by 5–10% across the experimental conditions (χ^2^ = 18.53, *P* < 0.01; mean ± SD: 1.47 ± 0.15, 1.41 ± 0.11, and 1.33 ± 0.12 m in the CT20, CT30, and CT40 conditions, respectively). This effect may be confirmed with a larger sample size (see Supplementary Table S1) when the participant #1 seems to be more affected than participant #2.

**Figure 3:**
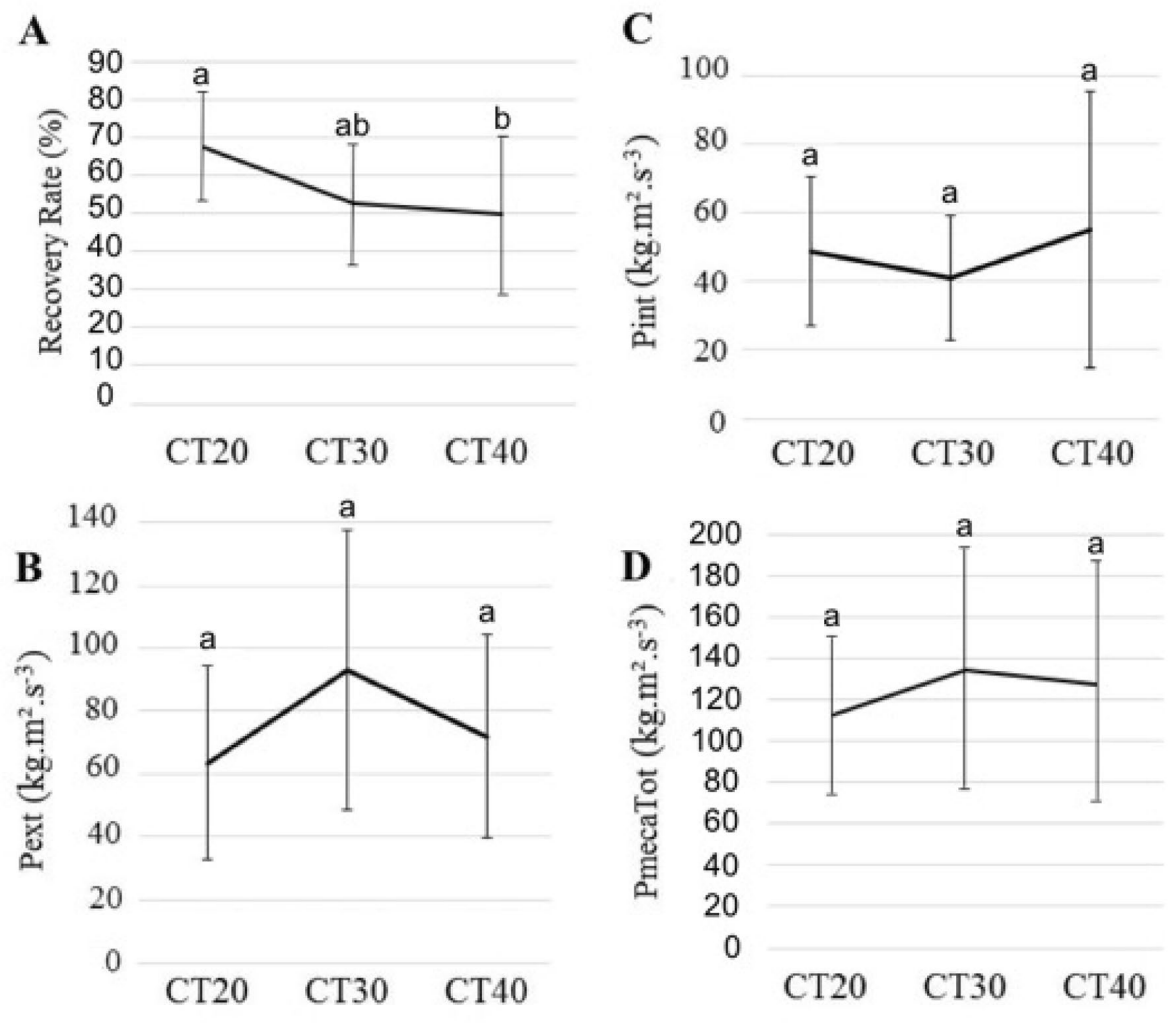
Energy recovered and powers. Mean (± SD) of the recovery rate (**A**), external power *P*_*ext*_ (**B**), internal power *P*_*int*_ (**C**)**,** and total mechanical power *P*_*mecaTot*_ (**D**) of the system formed by the two individuals plus the box they carry (PACS) in the CT20, CT30, and CT40 conditions (*N* = 9 pairs). The Recovery Rate is the percentage of energy recovered during one gait cycle by the PACS. *Pext, Pint*, and *PmecaTot* are respectively the positive external, internal, and total work per unit of time produced by the PACS. For the same variable the bars bearing different letters represent significantly different values (GLMM followed by a *post-hoc* test for multiple comparisons across conditions).

The pendulum dynamics of the CoM is an important characteristic of skilled walking^22,23^. In order to evaluate the pendulum-like behavior of the PACS, we compared the displacement of the CoM_PACS_ with that of an Inverted Pendulum System (IPS) model^10^. We found that the recovery rate (*RR*), which represents the percentage of conversion between kinetic and potential energy during walking^24^, tends to decrease from CT20 to CT30 and then to remain stable when load mass increased (Fig. 3A, χ^2^ = 5.45, *P* = 0.06; 67.07 ± 14.88, 52.40 ± 15.46, 49.70 ± 20.44% for CT20, CT30, and CT40 respectively). This tendency needs to be confirmed with a larger sample size (see Supplementary Table S1).

We also analyzed the external (Fig. 3B), internal (Fig. 3C), and total mechanical powers (Fig. 3D) produced by the PACS, and found that they did not differ across the experimental conditions (χ^2^ = 4.16, *P* = 0.12, χ^2^ = 1.30, *P* = 0.52, χ^2^ = 1.54, *P* = 0.46 respectively). Thus, the mechanical costs of the CoM_PACS,_ and of the body-segment movements were not affected by the load.

**Figure 4:**
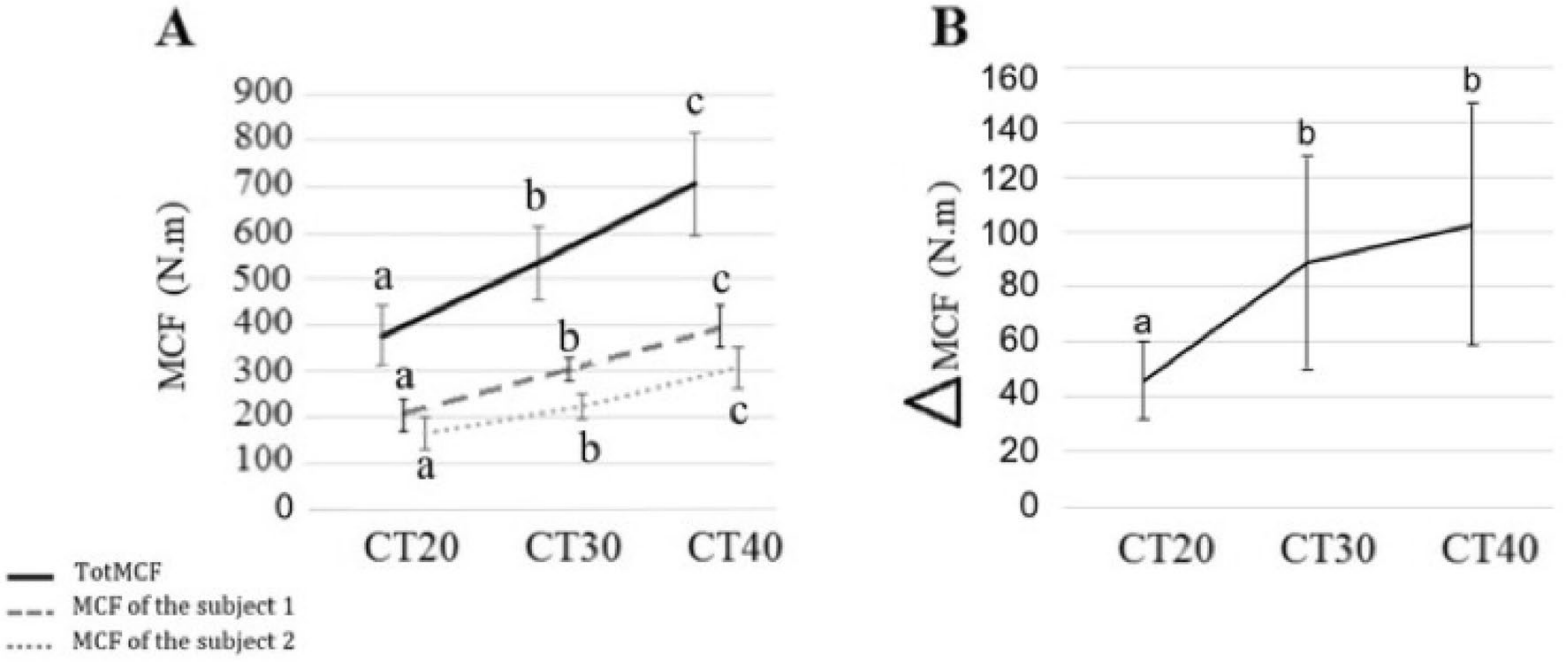
Moment cost function. Mean (± SD) of the total moment cost function (bold line: *TotMCF*, i.e., the sum of the MCF of participant #1 and #2), mean MCF of participant #1 (holding the load with the right hand, dashed line), and mean MCF of participant #2 (holding the load with the left hand, dotted line) (**A**)**.** Difference between the MCF of participant #1 and #2 (ΔMCF) (**B**)**.** The MCF corresponds to the sum of the joint moments applied to the arms, and is averaged over all gait cycles. For the same metric, the bars bearing different letters represent significantly different values (GLMM followed by a *post-hoc* test for multiple comparisons across conditions).

In addition, in order to quantify the internal efforts produced by the upper-limbs of the individuals during the transport of the load, we computed a moment cost function (MCF) as the sum of the upper limb joint moments. The total MCF (TotMCF) of the two individuals increased linearly with the load (Fig. 4A, χ^2^ = 55.71, *P* < 0.01), i.e., by 39.35% between CT20 and CT30 (z = 9.28, *P* < 0.01), by 30.14% between CT30 and CT40 (z = 9.92, *P* < 0.01), and by 81.22% between CT20 and CT40 (z = 19.20, *P* < 0.01,). A difference in MCF occurred systematically between the two individuals (ΔMCF), showing that participant #1 (holding the load with his right hand) produced greater joint moments than participant #2 (holding the load with his left hand) (Fig. 4B, χ^2^ = 17.18, *P* < 0.01). A post hoc analysis showed that ΔMCF increased by 95.15% between CT20 and CT30 (z = 3.92, *P* < 0.01and by 124.33% between CT20 and CT40 (z = 5.13, *P* < 0.01 but did not differ significantly between CT30 and CT40 (z = 1.20, *P* = 0.43).

Finally, a synergy analysis^13^ was performed in order to reveal the coordination structure for each participant, and across participants.

**Figure 5:**
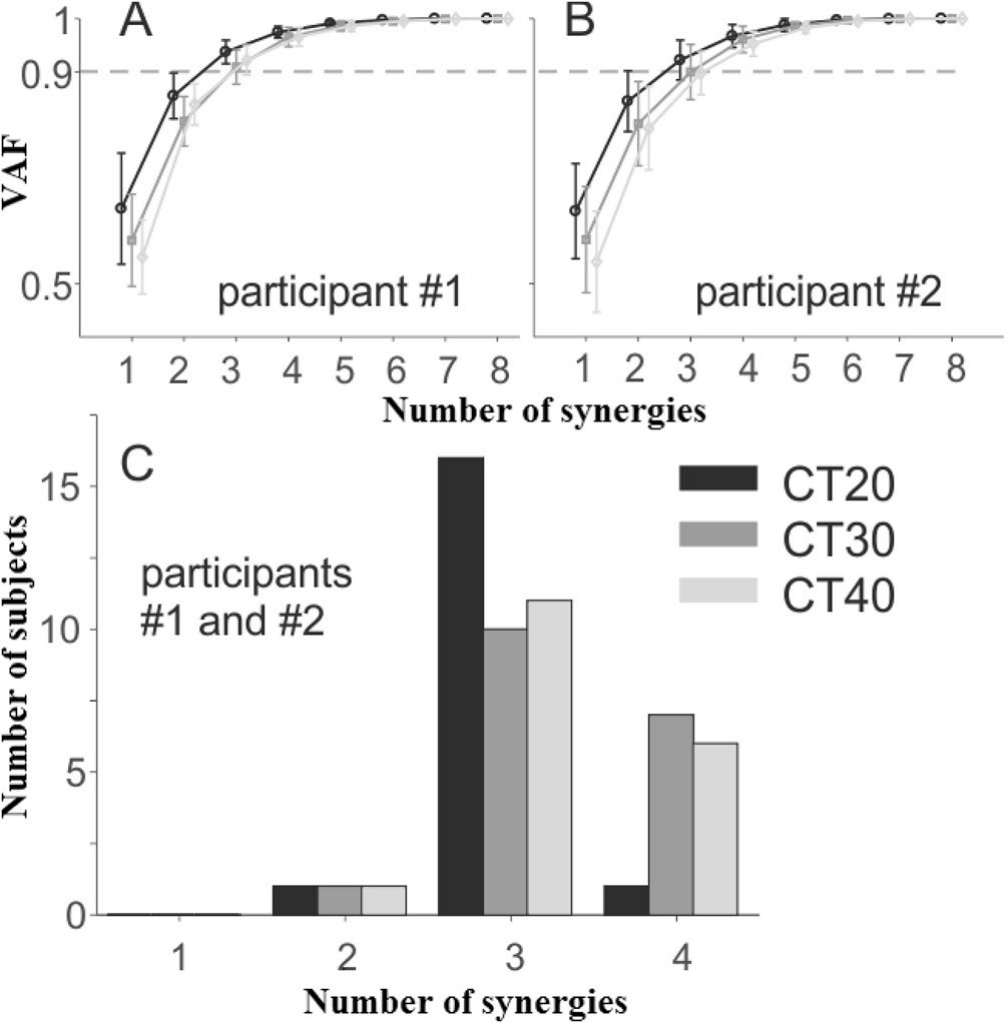
Cumulative variance and number of individual synergies in each experimental condition. Mean (± SD) of the Variance Accounted For (VAF) as a function of the number of synergies extracted for participant #1 (**A**), who holds the load with the right hand, and for participant #2 (**B**), who holds the load with the left hand. Frequency histogram corresponds to the number of synergies in each condition, as determined by the criterion “VAF > 90%” (**C**). Three synergies were selected for all participants in further analysis.

**Figure 6:**
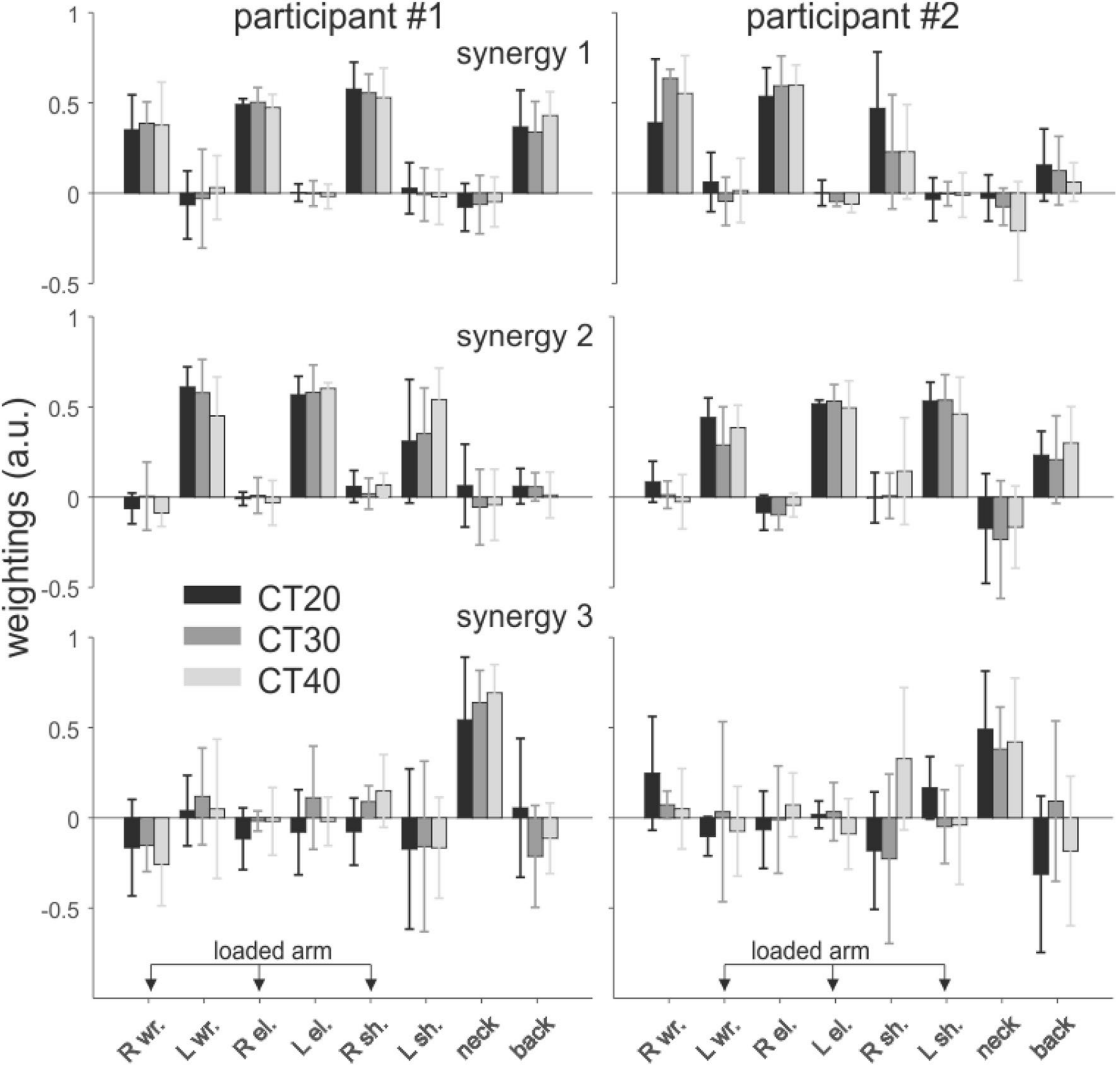
Individual synergy vectors. Mean (± SD, in arbitrary units) joint moment weightings of each synergy vector, for each participant, and across each condition. The vectors were normalized to unit length. **R** and **L** stand for right and left, respectively, **wt.** for wrist, **el.** for elbow, and **sh.** for shoulder. The loaded arm is indicated for each participant in the pair. In front view participants #1 and #2 were positioned on the right and left side of the load, respectively.

The frequency distribution of the number of synergies for each participant had a mode at *N* = 3 in each condition (Fig. 5C), and the number of synergies did not significantly differ across conditions (χ^2^ = 0.36, *P* = 0.83: 3.0 ± 0.3, 3.3 ± 0.6, and 3.3 ± 0.6 for the CT20, CT30, and CT40 conditions respectively). As a consequence, only three synergies were considered in the subsequent analysis. Figure 6 shows the average weightings of each joint moment for the three synergy vectors of each participant in the nine pairs studied. The first and second synergies show high weightings for the wrist, elbow and shoulder joint moments, either of the right side (synergy 1) or of the left side (synergy 2). Therefore, these joint moments co-varied during the collective transport task. The joint moments at the neck and back also co-varied with the joint moments at the wrist, elbow and shoulder, as suggested by their greater weightings. The third synergy was constituted mainly of the neck joint moment, meaning that it co-varied less consistently with the other joint moments. The changes in the weightings of the synergy vectors were not significantly affected by the experimental conditions. When comparing the synergy vectors across the different conditions the averaged Pearson’s *r*-values across all comparisons were 0.83 ± 0.14, 0.83 ± 0.13, and 0.44 ± 0.57 for synergy 1, 2, and 3 respectively, for participant #1 (right arm loaded), and 0.81 ± 0.19, 0.78 ± 0.17, and 0.29 ± 0.54 for synergy 1, 2, and 3 respectively, for participant #2 (left arm loaded). The *r*-values were consistent across conditions for the two participants (χ^2^ = 2.06, *P* = 0.36; χ^2^ = 0.69, *P* = 0.71; χ^2^ = 0.61, *P* = 0.73, for synergy 1, 2, and 3 respectively), showing that a similar coordination structure underlies the variation of the joint moments in the different conditions.

**Figure 7:**
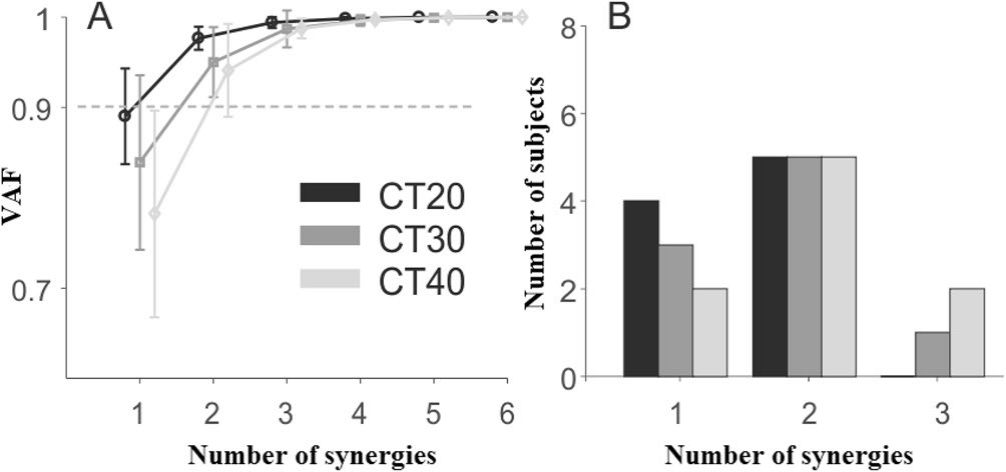
Cumulative variance and number of synergies for the paired participants in each experimental condition. Mean (± SD) of the Variance Accounted For (VAF) as a function of the number of synergies extracted from the joint moments of the loaded arms of the two participants in each pair at the same time (**A**). Frequency histogram illustrates the number of synergies in each condition, as determined by the criterion “VAF > 90%” (**B**).

**Figure 8:**
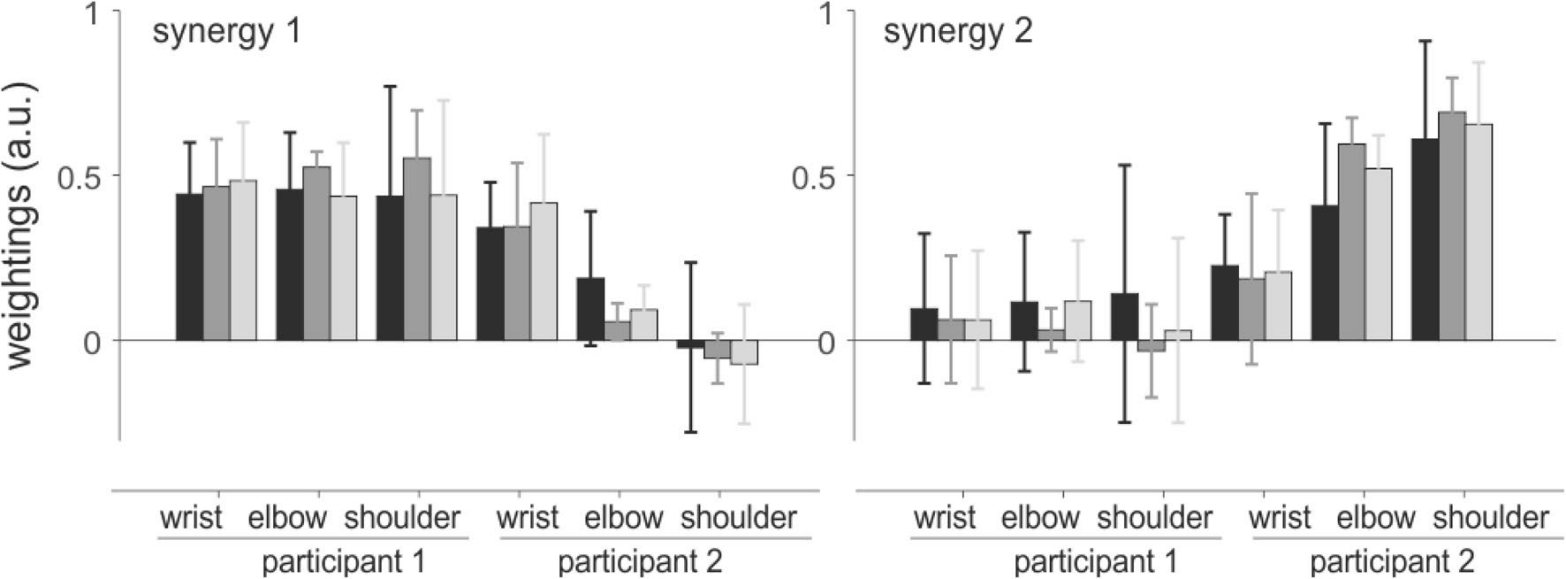
Synergy vectors of the pairs of participants. Mean (± SD, in arbitrary units) weightings of the synergy vectors extracted from the joint moments of the two participants of each pair at the same time. Only loaded arms were considered in this analysis. In front view participants #1 and #2 were positioned on the right and left hand side of the load, respectively.

The number of synergies of the loaded arms of the paired participants considered simultaneously was similar across conditions (χ^2^ = 0.50, *P* = 0.78), with average values of 1.6 ± 0.5, 1.8 ± 0.7, and 2.0 ± 0.7 in the CT20, CT30, and CT40 conditions, respectively (Fig. 7). Consequently, only two synergies were considered in the subsequent analysis. On the whole, synergy 1 comprised the joint moments of participant #1, and synergy 2 those of participant #2 (Fig. 8). The weightings of the wrist joint moment of participant #2 had similar vertical amplitude in both synergies, showing that this joint moment co-varied with the elbow and shoulder joint moments of both participants. Synergies were not affected by experimental conditions. The averaged Pearson’s *r*-values across conditions were 0.83 ± 0.21 and 0.81 ± 0.24 for synergy 1 and 2, respectively. There were no significant differences in *r*-values across conditions (χ^2^ = 1.07, *P* = 0.58; χ^2^ = 1.10, *P* = 0.58 for synergy 1 and 2 respectively), showing that the synergies were consistent across experimental conditions.

## Discussion

In this study, we hypothesized that the gait patterns of two individuals walking together while transporting a load could be altered through increasing the mass of the transported load. Results confirm our hypothesis. They also suggest that, despite the apparent symmetry of the task, the two individuals involved in a collective transport task do not have symmetrical roles, and that they may coordinate their behavior so that the mechanical energy of the system is kept constant. Overall, as discussed below, our data show that the biomechanics of a collective carriage task cannot be extrapolated from the results based on single carriers.

### Single versus collective task

Our results confirm the conclusion made in a previous study^10^: individuals are as energy-efficient when carrying a load together as when walking alone, for loads representing less than 30% of the sum of the body masses of the two participants. We noted, however, a tendency towards a deterioration of the efficiency of the locomotion with increasing load mass, as shown by the decrease observed in *RR* -percentage of energy recovered-. Nonetheless, the range of *RR* values found for the CoM_PACS_ in the three conditions tested,—i.e., ~ 50–67%—is in agreement with the values of *RR* found in previous studies on load carriage in humans, whether in pairs of individuals^10,11^ or in single individuals^25^. For example, Bastien *et al*^25^ found a maximum *RR* of 65% for Nepalese porters, and a *RR* between 61 and 64% for untrained individuals carrying loads representing 10–80% of their body mass. Thus, the displacement of the CoM_PACS_ of a pair of individuals carrying a load representing less than 40% of their total body mass exhibits the same pendular behavior as the displacement of the CoM of an individual walking alone and carrying a load of equivalent mass^25^. We also noted that the pairs performed the task at the same speed in all conditions, but that the vertical amplitude of the CoM_PACS_ oscillations decreased when load mass increased. No relation was therefore found between the speed and the vertical amplitude of the CoM_PACS_, a result consistent with previous studies in individual carriers^12^.

Finally, contrary to previous studies^4,5^ on single individuals where increasing load mass was found to increase step length (+ 13 cm per step with a 19% body mass increase in load mass), we observed a significant shortening of the step length of both participants when the load mass increased (− 7 cm per step with a 20% increase in load mass). Our hypothesis is that both the step length and CoM_PACS_ reductions could be explained by the collective nature of the task.

### Conservation of mechanical energy and power

We found that the internal, external and total mechanical power did not vary significantly when load mass increased. Surprisingly, each subject adapted its locomotion to make the whole PACS efficient, whatever the load carried. These results differ from those obtained regarding single individuals^1,4^ in which increasing load was found to trigger augmenting mechanical work. Consequently, it seems that the effect, on the energy of the system, of carrying a load amounting to a certain threshold is different for a single individual and for a pair of carriers. Since the participants were neither able to communicate orally during the experiments nor instructed to maintain the whole system mechanical power constant across conditions, the strategy they used was implicit and may represent an intuitive means to perform the task in a coordinated way. This strategy could be based on the observed reduction in step length. The decreasing in step length and in CoM_PACS_ vertical amplitude could be explained by the collective nature of the task. More precisely, the need to control a greater mass collectively could have been more challenging in terms of balance, or in terms of controlling the position of the load. Shortening the length of the steps may have helped to improve this control.

This adaptation in walking pattern induces different body mass velocities (linear and angular), which explains why the internal and total mechanical costs remain approximately the same across the three experimental conditions. According to the fundamental principle of dynamics we assume that the decrease in step length also impacts body mass accelerations and in turn affects ground reaction forces, lower-limb constraints, and their moment cost function. Unfortunately, this assumption founded on the laws of physics remains to be tested because our experimental setup did not allow us to assess the ground reaction forces under the four feet independently*.*

### Differential roles of the two carriers

We observed differences in the upper-limb internal efforts of the two participants: participant #1 (holding the load with the right hand) made greater efforts than participant #2 (holding the load with the left hand) in all conditions. These differences were observed in all pairs of individuals, irrespective of whether they were right- or left-handed. The reason why participant #1 made more efforts than participant #2 cannot be explained by an asymmetry of the carried object, or by a morphological difference between the two subjects –they were of similar height and weight. Rather, this result suggests an implicit choice made by each participant to haul more or less of the weight. This form of cooperative transport is reminiscent of what is observed in collective load carriage by ants, where some of the individuals in the group carry most of the load, while others are more in charge of guiding the movement^26^. Therefore, by a form of inverted anthropomorphism, we infer that participant #1 led the movement while participant #2 followed it. Note that this organization theoretically should lead to a mechanical optimum because no forces are applied against those of the partner. Indeed, the forces applied by both subjects are complementary, and the counteractions are avoided so that the system is mechanically efficient. The asymmetry observed in the efforts made may be limited by the maximal mechanical power produced by participant #1, and may depend on the mass of the load carried. Indeed, the difference in internal efforts between individuals within the same pair, when the load mass went from 20% up to 30% of the sum of the body masses, increased significantly but remained the same when the load carried went up from 30 to 40%. This suggests that the efforts were balanced between individuals and that a threshold existed for individuals to stabilize their coordination, around 30% of the sum of the body masses.

The synergy analysis reveals two “dynamic synergies” for each participant, corresponding to the wrist, elbow and shoulder joint moments (on the right side for the first synergy, and on the left for the second synergy), which did not vary with load mass. A third synergy, constituted mainly of the neck joint moment, was more variable across conditions. The structure of these synergies did not show any obvious trends with the increase in load mass. The consistency of the synergies in different conditions is coherent with the notion of linear synergy^14^ which states that the dynamics of the torques produced at each joint is a linear function of a single template torque function. Such template has been suggested to simplify the computation of the torques required during complex movements^27,28^. Previous studies have shown that these templates are robust in some experimental conditions (e.g., movement speed, or finger aperture during grasp movements^27,28^), but not in others (e.g., movement direction^29^). Hence, the invariant patterns found in the present study may reflect specific control strategies aimed at selecting the movements that require minimal changes in these patterns across conditions^30^.

The synergy analysis also shows that the joint moments of the two individuals were not completely independent. This was demonstrated by the presence of “mixed” weightings in the synergy vectors (Fig. 8). More precisely, the joint moments of the wrist of participant #2, who held the load with the left hand, co-varied with the wrist, elbow and shoulder joint moments of participant #1, who held the load with the right hand. The asymmetry between synergy 1 and 2 requires further investigation, but it is likely to be linked to the difference in joint constraint (ΔMCF) found between participant #1 and participant #2 (Fig. 4). Such coupling shows that the joint moments generated by the two subjects are not completely independent, i.e., that the wrist joint moments of participant#2 were partly locked to the joint moments generated by the arm of participant #1. It is important to note that this result does not contradict the results obtained when analyzing each subject independently, which shows that the wrist, elbow and shoulder joint moments co-varied in both participants (Fig. 6). In fact, the coupling between the wrist, elbow and shoulder joint moments of each participant can also be seen in Fig. 8. One could hypothesize that this (partial) synchronization between the joint moments of the two subjects may be purely mechanical. Yet, this interpretation would not explain the asymmetry observed in Fig. 8. Rather, this observation may reveal that the participants play a differential role. As discussed above, participant #1 may carry most of the load, with most of his arm joint moments co-varying with the wrist joint moments of participant #2. Finally, we found that the weight of the load had little influence on the inter-personal synergies.

### Conclusions

In conclusion, the present study shows that load mass did affect the gait characteristics of the system formed by two individuals and the load they carry. The center of mass vertical amplitude of the system formed by the pairs of participants with the load they carry tended to decrease when load mass increased, while their step length decreased, and walking efficiency tended to deteriorate. In addition, the total mechanical cost did not change when load increased, which is at odds with the result observed in single carriers. The analysis of joint moments suggests that the individuals composing the pairs may have distinct roles: one subject may guide the movement while the other may follow it. This implicit strategy may contribute to stabilize the total mechanical energy of the whole system, and may govern this particular type of interpersonal coordination.

## Methods

### Experimental protocol

Twenty healthy male adults participated in the study. To limit the effects of differences in the participants’ anthropometry, the volunteers were matched according to their height and weight.

The leg and hand dominances of each participant was noted before running the experiment. The subjects were placed at random on the left ((#1—right hand holding the load) or the right (#2—left hand holding the load) side of the carried object. It happened by chance that three lefthanders were affected to #2. The individuals had an average height of (mean ± SD) 1.77 ± 0.07 m (#1), and 1.77 ± 0.05 m (#2), and an average weight of 74.78 ± 9.00 kg (#1), and 74.54 ± 12.38 kg (#2). The load was symmetrical in shape, and its weight was evenly balanced between the participants who were positioned randomly with respect to the object transported to counteract the effect of a dominant side (Fig. 1A).

The study was carried out with healthy individuals who wrote their informed consent to participate in the experiment and to be filmed and photographed. The experiment was non-interventional, and the movements performed by the volunteers were no more risky than those they perform in daily activities. The study was approved by the Research Ethics Committee of the University of Toulouse, France (Number IRB00011835-2019-11-26-172, Université Fédérale de Toulouse IRB #1).

The instructions given to the volunteers were: “*Move the load together from point A to point B*” and “*Any* c*ommunication between you is forbidden during the experiment*”. Point A and point B laid 20 m apart. No explicit instruction was given as to how fast the volunteers should perform the task. The volunteers were tested with three conditions called CT20, CT30, and CT40 corresponding to a load representing on average 20%, 30%, and 40% of the sum of their body masses respectively. The three conditions were tested in random order for each pair. To avoid adaptations due to familiarization or learning, only one trial per pairs and condition was recorded.

### Kinematics and kinetics

Thirteen MX3, and TS40 Vicon cameras (Vicon©, Oxford) were used to capture the positions of ninety-one retro-reflective markers taped on the system formed by the paired individuals and the load they carry (hereafter called Poly-Articulated Collective System—PACS): 42 markers on each individual^31,32^, and seven on the load (Fig. 1A). The acquisition frequency was set to 200 Hz. In order to record the walking patterns of the individuals at a stable speed, and thus to exclude the acceleration and deceleration phases at the beginning and end of each trial, the calibrated volume (30m^3^) corresponded to the central part of the walkway. This covered about two steps. Concerning kinematic analysis, the PACS was reconstructed with the Vicon Nexus™ 1.8.5 software. Reconstruction was impossible for one pair of individuals who had lost one reflective marker. The two lateral handles on each side of the load were equipped with a 6-axis force sensor (Sensix®, France) (Fig. 1B), allowing to record the reaction forces and moments at a sampling frequency of 2000 Hz. The kinematic and kinetic measurement errors were 1 mm for 1 m for the positions (Vicon system) and ± 0.01 N for the forces (Sensix sensors), respectively. The sensors frames were located with the help of screwed reflective markers. The data were filtered with 4^th^ order Butterworth filters with a cut-off frequency of 5 Hz for the kinematic data, and of 10 Hz for the kinetic data. To ensure at least one complete walking cycle for each subject of a pair, the gait cycle of the PACS was defined from the first heel strike of individual #1 to the third heel strike of individual #2.

### COM determination and related parameters

The carried object, which constituted the 33th segment of the PACS, was built in aluminum and was therefore extremely rigid. It was completely symmetrical about its sagittal plane (Fig. 1B) and therefore its weight was evenly balanced between the participants. The object was equipped with a rod at its center where standard cast iron discs could be slid to increase its weight. The CoM of the object was determined at the intersection point of the vertical lines obtained by hanging the object without discs with a thread fixed at different positions. When the object was loaded, the position of its CoM was then adjusted by taking into account the added cast iron discs and by considering a homogeneous mass distribution inside the discs.

The De Leva Anthropometric table^33^ allowed us to estimate the mass m_i_ as well as the CoM of each segment i (CoM_i_) of the PACS, and thus to compute its global CoM (CoM_PACS_) as follows:

1 $${\varvec{G}}{\text{PACS}} = \frac{1}{{m_{PACS} }}\mathop \sum \limits_{i = 1}^{n = 33} m_{i} {\varvec{G}}_{i}$$

with ***G***_***PACS***_ corresponding to the 3D position of the CoM_PACS_ in the frame R (the global coordinate system), *m*_*PACS*_ to the mass of the PACS, *n* the number of PACS segments (i.e. 16 segments per volunteer, plus one segment for the box), and ***G***_***i***_ corresponding to the 3D position of the CoM_i_ in R.

The vertical amplitude (*Az* = *Z*_max_ − *Z*_min_, in meters) of the CoM_PACS_ trajectory along two consecutive steps, and the length of two consecutive steps by each individual were also computed.

### Assessment of energetic exchanges

To assess energetic exchanges, forward kinetic work, vertical work, and external work of the forces applied to the CoM_PACS_ were computed^25^.

Forward kinetic work (*W*_kf_) was defined as the positive work needed to move the CoM_PACS_ forward, and it was calculated as the sum of the increments of forward kinetic energy (*E*_kf_) along the time curve:

2 $$E_{{{\text{kf}}}} = { }\frac{1}{2} m \overrightarrow{V_{f}}\left( {\text{t}} \right)^{{2}} _{/R}$$

with *m* being the mass of the individual, and $\overrightarrow {{V_{f} }}$ (t)_/R_ the linear forward velocity of the CoM_PACS_ in the frame R. The x-, y- and z-axis of the frame R, corresponding to the medio-lateral, antero-posterior, and vertical directions respectively, are illustrated in Fig. 1A.

Vertical work (*W*_v_) was defined as the positive work needed to move the CoM_PACS_ against gravity, and it was calculated as the sum of the increments of the vertical kinetic energy (*E*_kv_) plus the potential energy (*E*_pot_) along the time curve with:

3 $$E_{{{\text{kv}}}} = { }\frac{1}{2} m \overrightarrow{V_{v}}\left( {\text{t}} \right)^{{2}} _{/R}$$

and

4 $$E_{{{\text{pot}}}} = mgh_{{/{\text{R}}}}$$

where $\overrightarrow {{V_{v} }}$ (t)_/R_ is the linear vertical velocity of the CoM_PACS_ in R, *g* = 9.81 m s^−2^ is the acceleration due to gravity, and *h*_/R_ is the height of the CoM_PACS_ in R.

The external work (*W*_ext_), corresponding to the positive external work needed to raise and accelerate the CoM_PACS,_ was computed as the sum of the increments of the external mechanical energy (*E*_ext_) along the time curve with:

5 $$E_{{{\text{ext}}}} = E_{{{\text{pot}}}} + E_{{{\text{kv}}}} + E_{{{\text{kf}}}}$$

The energy recovered (called recovery rate (*RR*)^10^) by the CoM_PACS_ in the sagittal plane was computed with the following formula^17^:

6 $$RR = { 1}00\frac{{W{\text{kf}} + W{\text{v}} - W{\text{ext}}}}{{W{\text{kf}} + W{\text{v}}}}$$

*RR* is the percentage of kinetic energy converted into potential energy^7,24,25,34,35^ and vice versa.

In the present study, internal work was also considered in order to encompass the coordination between all body segments. Based on the assumption of a conservative Poly-Articulated Model (PAM), internal work (*W*_int_) was computed as the sum of the increments of the *E*_int,k_ along the time curve with:

7 $$E_{{{\text{int}},{\text{k}}}} = \frac{1}{2}~\mathop \sum \limits_{{i = 1}}^{{33}} (m_{i} \overrightarrow {{V_{{~i}} }} \left( {\text{t}} \right)^{{\text{2}}} _{{/{\text{R}}*}} + m_{{\text{i}}} K_{{i^{2} }} {\text{ }} \times \vec{\omega }^{2} _{i} /_{{{\text{R}}*}} )$$

where *m*_i_ is the mass of the ith segment, $\overrightarrow {{V_{i} }}$(t)_/R*_ the linear velocity of its CoM in the sagittal plane of the barycentric coordinate system (R*), *K*_i_ its radius of gyration around its CoM, and $\vec{\omega }_{i}$^2^_/R*_ its angular velocity in R* ^36^.

The total mechanical energy of the PACS (*E*_tot_) was computed as the sum of the internal kinetic energy (*E*_int,k_) of each segment, plus the potential energy (*E*_pot_), and the forward (*E*_kf_ ) and vertical (*E*_kv_ ) kinetic energy of the CoM_PACS_ in the sagittal plane^21,25,37,38^:

8 $$E_{{{\text{tot}}}} = E_{{{\text{int}},{\text{k}}}} + E_{{{\text{pot}}}} + E_{{{\text{kf}}}} + E_{{{\text{kv}}}}$$

Finally, the total mechanical power (*P*_mecaTot_) was used to assess the amount of energy spent or gained by the CoM_PACS_ per unit of time (*Δt*):

9 $$P_{{{\text{mecaTot}}}} = \frac{{W{\text{ext}}}}{\Delta t} + \frac{{{ }W{\text{int}}}}{\Delta t} = P_{{{\text{ext}}}} + P_{{{\text{int}}}}$$

### Calculation of internal efforts

The resultant joint moments at the wrist, elbow, shoulder, neck, and back joints were calculated using a bottom-up Newton–Euler recursive algorithm^39^. Cardanic angles were used to represent the rotation of the segments coordinate system relative to the global coordinate system^40^. The segment masses, inertia tensors, and center of mass locations were estimated for each subject according to the scaling equations proposed in Dumas et al. (2007)^41^. In order to estimate the muscular torque produced at all the joints of the upper-limbs, shoulders, neck, and back, the Moment Cost Function (*MCF* in kg m^2^ s^−2^, ^42^) was computed as follows:

10 $${\text{MCF}} = \sqrt {M_{L\_wt}^{2} } + \sqrt {M_{R\_wt}^{2} } + \sqrt {M_{L\_el}^{2} } + \sqrt {M_{R\_el}^{2} } + \sqrt {M_{L\_sh}^{2} } + \sqrt {M_{R\_sh}^{2} } + \sqrt {M_{back}^{2} } + \sqrt {M_{neck}^{2} }$$

where M_L_wt_, M_R_wt_, M_L_el_, M_R_el_, M_L_sh_, M_R_sh_, M_back,_ and M_neck_ are the mean values over a PACS gait cycle of the three-dimensional left and right wrist, left and right elbow, left and right shoulder, top of the back and neck moments, respectively. $\sqrt {{\text{M}}^{2} }$ represents the Euclidian norm of M, i.e. $\sqrt {\sum\nolimits_{i = 1}^{3} {\left( {M_{i} } \right)^{2} } }$, with M_i_ the i-th component of the vector M.

We summed the *MCF* values of the two individuals of each pair to obtain the total moment cost function (TotMCF). The TotMCF allows to quantify the global muscular effort developed at the upper-limbs of the PACS during one gait cycle of the carrying of the load. The *MCF* difference (∆*MCF*) between the two individuals was also computed to investigate whether the volunteers developed the same efforts while carrying the object.

### Kinetic synergy analysis

We extracted the synergies by using a principal component analysis (PCA) applied to the wrist, elbow, shoulder, back, and neck joint moment on the right and left sides of the body. The PCA was used to reduce data dimensionality^13,35,43^. It consisted in the eigen-decomposition of the co-variance matrix of the joint moment data (Matlab *eig* function). The joint moments data were arranged in time × joint moment matrices. We called the eigenvectors extracted from the PCA synergy vectors^13^. The number of synergies was determined from the VAF (Variance Accounted For), which corresponds to the cumulative sum of the eigenvalues, ordered from the greatest to the lowest value, normalized by the total variance computed as the sum of all eigenvalues. We defined the number of synergies as the first number for which the VAF was greater than 0.9. The synergy vectors retained were then rotated using a Varimax rotation method to improve interpretability^44^.

We extracted the synergy vectors for each experimental condition separately. We first performed an analysis on each individual separately. In this analysis the initial data matrices were constituted of all available time frames in line, concatenated, and of eight columns corresponding to each joint moment, namely the right wrist, left wrist, right elbow, left elbow, right shoulder, left shoulder, back, and neck. The values in the matrix corresponded to the norm of the joint moment vector at a given time frame. We then performed a second analysis to identify possible co-variations between the joint moments of the two participants in each pair. The columns of the initial matrices were thus constituted of the joint moments of the two loaded arms, i.e., the right wrist, elbow, and shoulder joint moments of participant #1, plus the left wrist, elbow and shoulder joint moments of participant #2. The synergy vectors were compared across conditions by computing Pearson’s r correlations on their PCA weightings, after being matched together, also using Pearson’s r to identify the best matches.

### Statistical analysis

We used generalized linear mixed models (GLMM)^45^ to compare the velocity and the vertical amplitude of the CoM_PACS_, the length and duration of the gait cycles, the recovery rate, the external, internal, and total mechanical power produced by the PACS, the TotMCF and ∆MCF, the number of synergies, as well as the Pearson’s r-values across conditions.

The experimental condition was entered as a fixed factor in the model, and individuals as a random variable. We used a Gaussian GLMM for all variables, except for the comparison of the number of synergies across conditions, for which a Poisson GLMM was used. For Gaussian GLMMs we systematically inspected the normality of the model residuals with Q-Q plots. We used the functions *lmer()* and *glmer()* of the R package *lme4* ^46^ to run the Gaussian and the Poisson mixed models, respectively. The effect of experimental conditions was tested by comparing the deviance of the model with and without the fixed factor with a χ^2^ test. Multiple comparisons across experimental conditions were performed with the function *glht()* of the *multcomp* R package^47^ using the default Tukey test as *post-hoc* test. Pearson’s *r* were Fisher Z-transformed before running the analyses. The significance threshold was set to 0.05. All data in the text are given as mean ± SD. Since our sample size was low, which could lead to inflate the Type II error (not rejecting H_0_ when H_0_ is false), we followed the recommendations of Nakagawa & Foster (2004)^49^ and provide in the Supplemental Table S1 the value of Cohen *d* standardized effect size^50^, along with its 95% confidence interval^51^, for each studied parameter and each paired comparison between conditions. A confidence interval that largely extends on both sides of zero indicates an absence of effect that would probably not change with increasing the sample size.

### Ethics statement

All methods used in this study were carried out in accordance with relevant guidelines and regulations.

## Supplementary information

Supplementary Information.
